# Pattern formation in thin polymeric films *via* electrohydrodynamic patterning

**DOI:** 10.1039/d2ra01109c

**Published:** 2022-03-28

**Authors:** Guowei Lv, Hongmiao Tian, Jinyou Shao, Demei Yu

**Affiliations:** School of Chemistry, State Key Laboratory of Electrical Insulation and Power Equipments, MOE Key Laboratory for Non-Equilibrium Synthesis and Modulation of Condensed Matter, Xi'an Jiaotong University Xi'an 710049 Shaanxi P. R. China dmyu@xjtu.edu.cn; Xi'an Aerospace Chemical Propulsion Co., Ltd. Xi'an 710025 Shaanxi P. R. China; State Key Laboratory of Manufacturing Systems Engineering, Xi'an Jiaotong University Xi'an 710049 Shaanxi P. R. China

## Abstract

The free surface of a thin polymeric film is often unstable and deforms into various micro-/nano-patterns under an externally applied electric field. This paper reviews a recent patterning technique, electrohydrodynamic patterning (EHDP), a straightforward, cost-effective and contactless bottom-up method. The theoretical and numerical studies of EHDP are shown. How the characteristic wavelength and the characteristic time depend on both the external conditions (such as voltage, film thickness, template-substrate spacing) and the initial polymer properties (such as rheological property, electrical property and surface tension) is theoretically and experimentally discussed. Various possible strategies for fabricating high-aspect-ratio or hierarchical patterns are theoretically and experimentally reviewed. Aligning and ordering of the anisotropic polymers by EHDP is emphasized. A perspective, including novelty and limitations of the methods, particularly in comparison to some conventional patterning techniques, and a possible future direction of research, is presented.

## Abstract 2

A review about electrohydrodynamic patterning (EHDP), including origin, theoretical and numerical studies, pattern formation process, fabrication of hierarchical structures, aligning and ordering of the anisotropic polymers and perspective.

## Graphical abstract

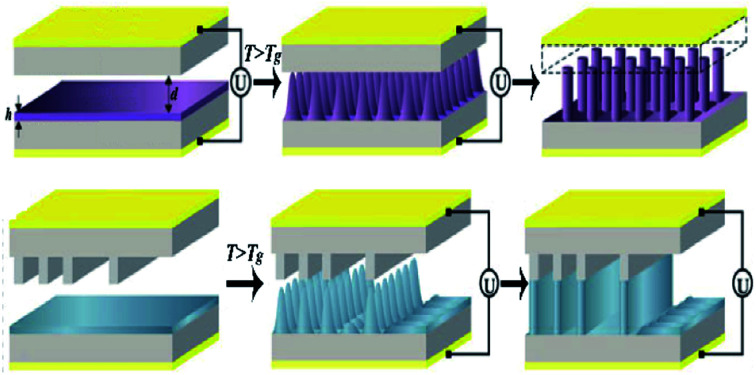

## 1 Origin of EHDP

Over the past few decades, the urgent need of micro- and nano-structures for manufacturing high-performance devices, such as integrated circuits, optoelectronic devices, and sensor arrays, has motivated the development of new patterning techniques.^1–5^ In 1999, Chou *et al.* observed that a periodic pillar array with a micrometer scale was formed in a thin polymer film.^6–14^ Although without the external electric field, the key driving force for the formation of the micro-/nano-structures is the internal localized electrical field generated between the contactor and the polymer film. In 2000, Schaffer *et al.* reported a straightforward, cost-effective and contactless (positive replica) patterning technique, electrohydrodynamic patterning (EHDP), that creates and replicates lateral hierarchical structures with a submicrometre length scale on various kinds of materials (*e.g.* thermoplastic polymer,^15–22^ thermosetting polymer,^23–29^ photocurable resin,^30–34^ ceramic,^35–37^ and so on) under an externally applied electric field.^38–47^ Especially, the capability of EHDP with a featureless template to fast and economically create large-scale three-dimensional micro-scale structures, even though no long range order, is particularly attractive because the difficulty, time, and cost in designing and making pre-patterned templates limit the flexibility and wide application of conventional lithography, especially when large numbers of different patterns are to be fabricated.^48^ It is worth noting that a higher value of *f* (*h*/*H*, the ratio of the polymer film thickness to the template-substrate spacing) leads to a denser packing of the polymer columns and to an enhanced electrostatic repulsion between the equally charged columns, resulting in columns with a perfect hexagonal symmetry and, however, an accelerating coarsening.,^15^ a lower value of *f* leads to the suppression of coarsening.^49,50^ Hence, the highly ordered arrays of columns only can be obtained in a very narrow *f* range.^51^ In addition, EHDP just need a minimized external force to maintain a proper air gap between the liquid polymer and the template, avoiding a poor geometrical integrity of the duplicated micro-/nano-structure or even to an irreversible damage of the substrate and the template.^52^

**Fig. 1:**
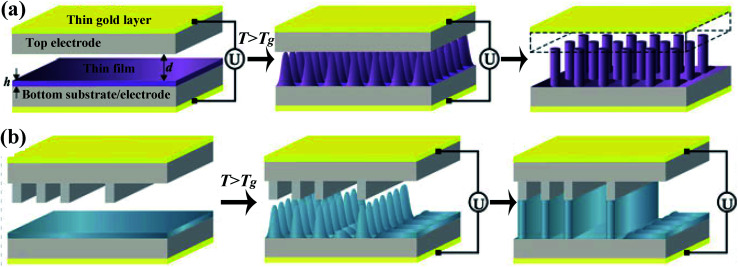
The diagrammatic sketch of EHDP with (a) a featureless template and (b) a patterned template. Reproduced with permission.^66^ Copyright 2013, Springer International Publishing Switzerland.

The diagrammatic sketch of EHDP is shown in Fig. 1.^53^ Firstly, a thin film of the polymer is spin-coated on a conductive substrate (bottom electrode). Then a template (top electrode) is put on the top of the polymer film, leaving an air gap. Secondly, the assembly is then thermally maintained above the glass transition temperature (*T*_g_) of the polymer, and then an external voltage is applied between the bottom and top electrode. This applied voltage causes a destabilizing electrostatic force due to the mismatch of the dielectric constant or the electrical conductivity between polymer and air. Once the destabilizing electrostatic force overcomes the stabilizing surface tension and the viscous resistance, the flat polymer surface will be destabilized and forced to flow upward to the top electrode. The growth of peaks reduces the air gap between the polymer film and the template, which strengthens the electrical field and accelerates the evolution. As the peaks grow, the capillary pressure also increases. Therefore, the competition between destabilizing electrostatic pressure and the stabilizing capillary pressure selects a maximum characteristic wavelength *λ* and a characteristic time *τ*.^48^ Finally, the micro-/nano-structure of the polymer will fully contact with the top electrode and then reach a steady state. After that, a slow cooling of the assembly to room temperature or UV light cures the micropillars array. In this process, the template can be either featureless (Fig. 1a),^15,17,27^ generating an array of periodic pillars, or patterned (Fig. 1b),^15,37,39,41,46,54–65^ forming a positive replica of the template.

## 2 Theoretical and numerical studies

The overall pressure distribution at the viscous film surface is

1 *p*(*h*) = *p*_0_ + *p*_vdW_(*h*) + *p*_*γ*_(*h*) + *p*_e_(*h*)

where *p*_0_ is the atmospheric pressure, *p*_vdW_(*h*) is the disjoining pressure (arising from dispersive van der Waals interactions), *p*_*γ*_(*h*) is the Laplace pressure (stems from the surface tension *γ*) and *p*_e_(*h*) is the electrostatic pressure.^16^

For high enough values of electric field intensity, only the Laplace and electrostatic terms need to be considered. In a stability analysis, a small sinusoidal perturbation of the interface with wave number *q*, growth rate *ω*, and amplitude *u* is considered: *h*(*x*, *t*) = *h*_0_ + *u*e^*iqx*+*tω*^. The modulation of *h* gives rise to a lateral pressure gradient inside the film, inducing a Poiseuille flow *j*2
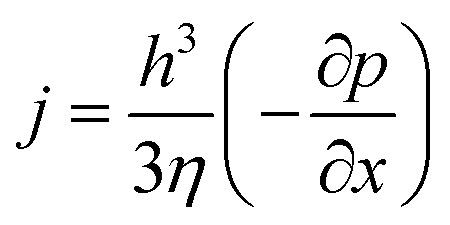
where *η* is the viscosity of the liquid. A continuity equation enforces mass conservation of the incompressible liquid3
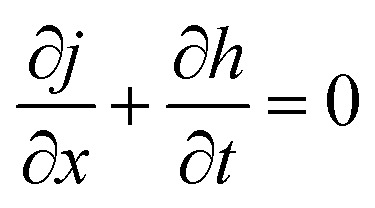


Eqn (1)–(3) establish a differential equation that describes the dynamic response of the interface to the perturbation. In a linear approximation, a dispersion relation is obtained4
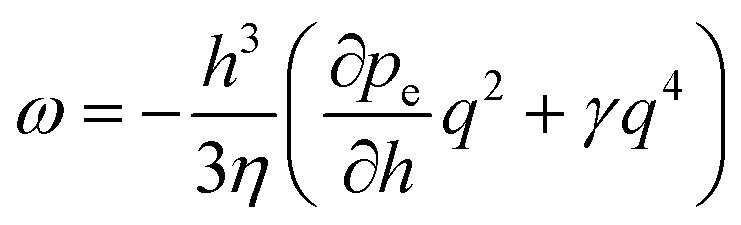


As opposed to the inviscid, gravity-limited case (*ω* ∝ *q*), the viscous stresses lead to a *q*^2^-dependence of *ω* in the long-wavelength limit, typical for dissipative systems. Fluctuations are amplified if *ω* > 0. With time, the fastest growing fluctuation will eventually dominate5
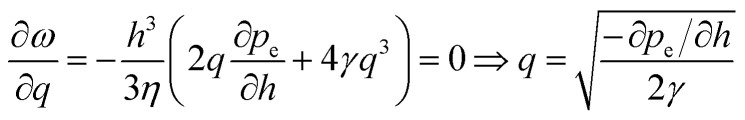


When mobile free charge is absent (*i.e.* perfect dielectric), the characteristic wavelength *λ* and the characteristic time *τ* is6
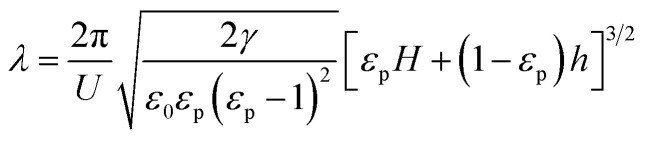
and7
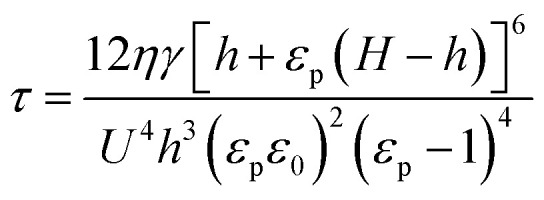
where *ε*_0_ is the dielectric constant of free space, *ε*_p_ is the dielectric constant of the polymer film, *γ* is the surface tension of the polymer film, *U* is the applied voltage, *h* is the thickness of the polymer film, and *H* is the template-substrate spacing.

When mobile free charge is present (*i.e.* leaky dielectric), the dielectric constant and a dimensionless conductivity parameter *S* jointly play roles. The latter represents a ratio of the process time scale to the time for charge conduction (*ε*_g_*ε*_0_/*σ*)

8 *S* = *σηγH*^3^/*ε*_0_^3^*U*^4^

Especially, when *S* ≫ 1, the characteristic wavelength *λ* and the characteristic time *τ* is9
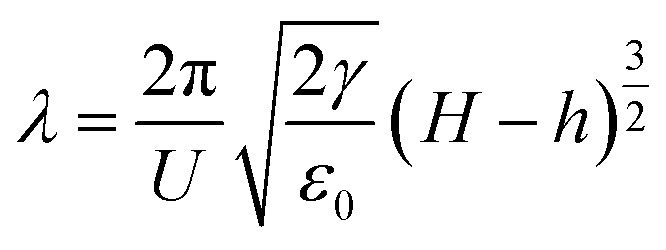
and10
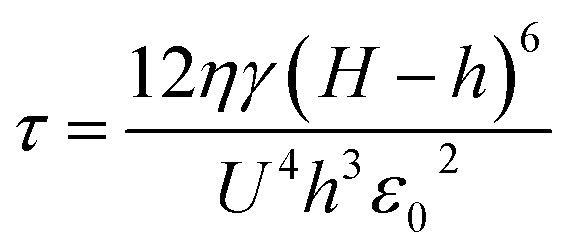
where *σ* is the electrical conductivity of the thin polymer film.^67–70^

## 3 Effect of the external conditions on the pattern formation

In continuing the steady development of integrated circuit-related fabrication, the ability to rapidly pattern polymers into smaller feature size and/or higher aspect ratio in order to realize devices with enhanced performance or even wholly new properties begins to take a more prominent role in their advanced applications.

**Fig. 2:**
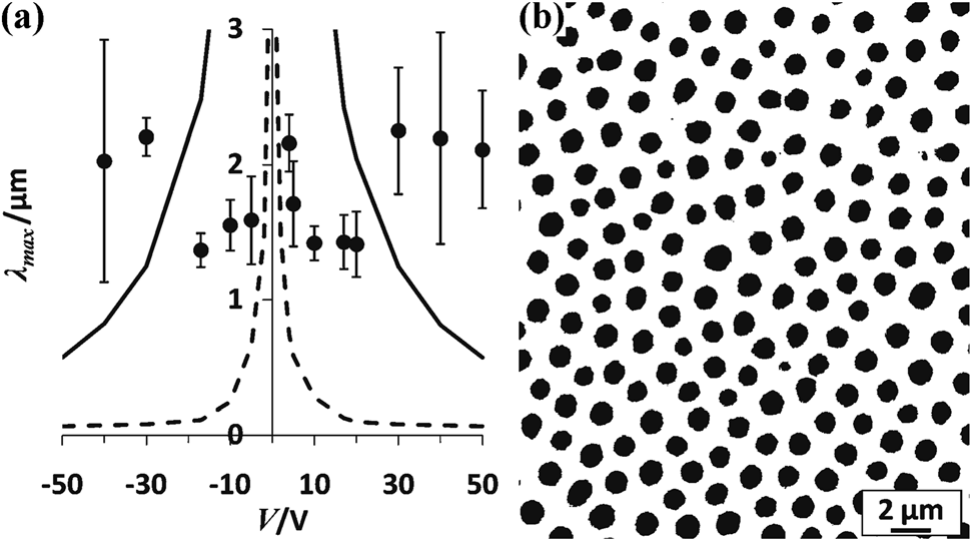
EHDP of PMMA2K-air. (a) Plot of the characteristic wavelength *λ vs.* the applied voltage. (b) An optical micrograph of pillars formed at 10 V. Reproduced with permission.^71^ Copyright 2011, American Chemical Society.

According to eqn (6)–(10), changing the external conditions such as decreasing the template-substrate spacing (*H*), increasing the polymer film thickness (*h*) and increasing the applied voltage (*U*) can fabricate micro-/nano-patterns with a shorter characteristic time and a smaller characteristic wavelength *via* EHDP for both the perfect and leaky dielectric. For example, Russel *et al.* showed that the characteristic wavelength *λ* decreases with the increase in the applied voltage (below 10 V) at a given system, as shown in Fig. 2a.^71^ However, the characteristic wavelength *λ* no longer decreases or even increases with the increase in the applied voltage (above 10 V) due to the dielectric breakdown in at least one of the layers. If layers break down, submicrometer features are unlikely, as shown in Fig. 2b. Hence, though theory predicts that changing the external conditions will decrease the characteristic wavelength *λ* to nanoscale level, the limit is set by the dielectric field strength of the layers. In addition, increasing the pattern growth velocity not only shortens the patterning time but also exhibits enhanced scalability for replicating small and geometrically diverse features.^72^

## 4 Effect of the polymer properties on the pattern formation

In EHDP, polymer film materials have played a critical role, since some limitations in accessing better-performance EHDP, such as higher efficiency, smaller feature size, and greater aspect ratio, are of material origin; moreover, the functionality of the generated EHD patterns is governed by EHD materials owing to the interesting properties these materials can offer. Hence, the performance of EHDP depends on the comprehensive properties of the polymer film to a certain extent.

### 4.1 Rheological property

**Fig. 3:**
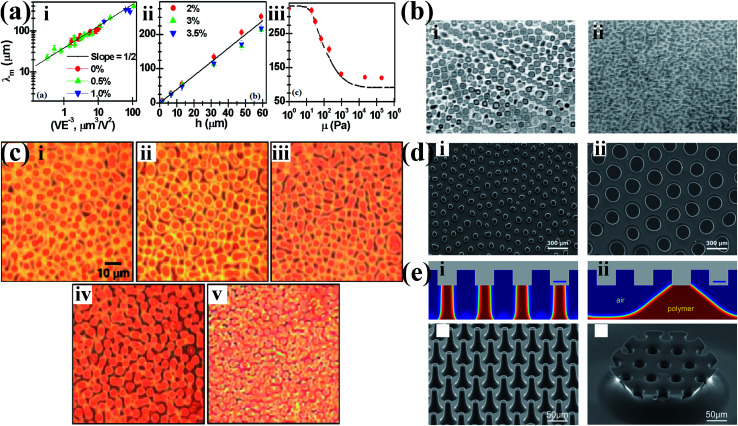
(a(i)) The characteristic wavelength *λ vs. VE*^−3^ in log–log scale for viscoelastic liquidlike films. (ii) The characteristic wavelength *λ vs.* the film thickness *h* for viscoelastic solidlike films. (iii) The characteristic wavelength *λ vs.* the storage modulus *μ* for viscoelastic films between the liquidlike and the solidlike regimes. Reproduced with permission.^24^ Copyright 2009, American Physical Society. (b) Optical microscopy image of EHDP patterns of (i) a thin liquid film of PI and (ii) a bilayer of PI and ODMS. Reproduced with permission.^77^ Copyright 2001, American Institute of Physics. (c) EHDP patterns of various Au(PS) thin films: (i) Au(PS) 0 vol%; (ii) Au(PS) 0.05 vol%; (iii) Au(PS) 0.25 vol%; (iv) Au(PS) 0.5 vol%; (v) Au(PS) 1.0 vol%. Reproduced with permission.^78^ Copyright 2008, American Chemical Society. (d and e) Simulated and experimental deformation of EHDP with (d) a featureless template and (e) a structured template for (i) the leaky dielectric and (ii) perfect dielectric. Reproduced with permission.^33^ Copyright 2016, Royal Society of Chemistry. Reproduced with permission.^31^ Copyright 2014, American Chemical Society.

The rheological property of the polymer noticeably governs the characteristic wavelength *λ* and the characteristic time *τ* of EHDP.^23–27,73–75^ Sharma *et al.* has systematically studied the role of the viscoelastic property of the polymer film in the pattern formation of EHDP.^24^ The viscoelastic films behaving like a liquid display long wavelengths governed by applied voltage (or electrostatic pressure) and surface tension, independent of its elastic storage and viscous loss moduli (Fig. 3a(i)); the viscoelastic films behaving like a solid display wavelengths always scales as ∼4 × film thickness, independent of its surface tension, applied voltage, loss and storage moduli (Fig. 3a(ii)); and the viscoelastic films behaving in a narrow transition zone between the liquidlike and the solidlike regimes display a wavelength governed by the storage modulus (Fig. 3a(iii)). It is interesting to note that the viscosity of the polymer film influences the characteristic time *τ* of EHDP. This offers an advantage in EHDP for decoupling time by varying the types or the molecular weight of polymers. For example, Dickey *et al.* showed that the photocurable systems (*e.g.* thiol vinyl ether, vinyl ether, thiol acrylate, DMS acrylate, epoxy) with a low viscosity (0.1–1.8 Pa s) formed pillar arrays nearly instantaneously under ambient conditions.^30,76^ Goldberg-Oppenheimer *et al.* showed that the use of the polymers with a low-viscosity (0.073–1.032 Pa s) evidently reduced the characteristic time.^19^ It is worth noting that the rheology property of the polymer is closely related to its temperature. For instance, Cheng *et al.* showed a faster growth of the surface patterns of polystyrene film at a higher temperature due to the lower viscosity.^20^ However, the molding temperature cannot exceed the decomposition temperature of the polymer in the pattern formation process.

### 4.2 Surface/interface tension

The decrease of the surface/interface tension of the polymer film is advantageous to fabricating the micro-/nanostructures with a smaller characteristic wavelength *λ* and a shorter characteristic time *τ*.^75,77,79–85^ For example, a clear reduction in length scale (2 times) and time scale (50 times) of polyisoprene (PI) film is observed by substituting the air gap with oligomeric dimethylsiloxane (ODMS) film, as shown in Fig. 3b. However, there is a trade-off by replacing air with another fluid because it may decrease the dielectric contrast difference of the system.^48^

### 4.3 Electrical property

#### 4.3.1 Dielectric constant

According to the linear stability analysis based on the perfect dielectric model, when free charges absent (*i.e.*, the perfect dielectric), the dielectric constant *ε*_p_ of the polymer film is important. A smaller characteristic wavelength *λ* and a shorter characteristic time *τ* is expected with increasing the dielectric constants *ε*_p_ of the layers.^78,86^ For instance, Bae *et al.* demonstrated that the increased dielectric constant of the polymer film by the incorporation of nanoparticles into the polymer film led to a significant reduction in the characteristic wavelength *λ*, as shown in Fig. 3c.^78,86^ However, the enhancement of the dielectric constant by loading nanoparticles is also limited as the excessive filler loading would result in the increase in the viscosity of the polymer and the undesirable nanoparticle agglomeration in the polymer film.

#### 4.3.2 Electrical conductivity

Particularly interesting is the characteristic wavelength *λ* and the characteristic time *τ* that is predicted from leaky dielectric model is smaller than that from the perfect dielectric model.^87–92^ Lv *et al.* demonstrated that a leaky dielectric could fulfill high-performance EHDP with a featureless template.^32–34^ A significant reduction in the characteristic wavelength *λ* (from 325.6 ± 14.7 μm to 154.5 ± 15.3 μm) and the patterning time (from 5 s to ≪1 s) are observed, as shown in Fig. 3d. Furthermore, according to the perfect and leaky dielectric model, the theoretical characteristic wavelength *λ* is 368 μm (the perfect dielectric) and 150 μm (the leaky dielectric), respectively, hence, the experimental results herein demonstrate a convincing experimental distinction between the ‘‘perfect’’ and ‘‘leaky’’ dielectric models in spite of a slight mismatch between the theoretical and experimental values especially from the perfect dielectric model. Moreover, Tian *et al.* theoretically and experimentally demonstrated that a leaky dielectric could significantly improve the aspect ratio of the micro-/nano-structures fabricated by EHDP with a structured template compared to a perfect dielectric, as shown in Fig. 3e.

**Fig. 4:**
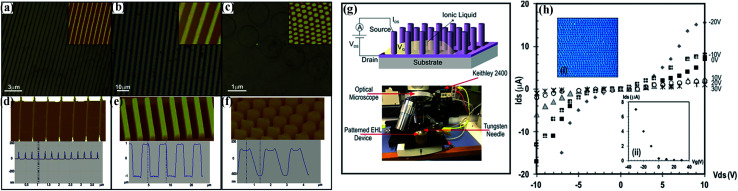
(a–f) Optical microscopy images with height AFM images (inset) and three-dimensional AFM micrographs with cross section analysis of EHDP patterns of PPy. (g) Schematic representation (top) and an overview image (bottom) of the configuration of a liquid-ion gate vertical FET using the EHDP patterns on the top of a Si–SiO_2_ substrate. (h) Drain current *versus* drain voltage characteristics of PPY electrolyte-gated transistor based on (i) EHDP pillars shown in a top-view optical image and (ii) gate voltage performance of the PPy-FET. Reproduced with permission from https://pubs.acs.org/doi/10.1021/acsnano.6b01246.^100^ Copyright 2016, American Chemical Society (notice: further permissions related to the material excerpted should be directed to the ACS).

Especially, the conducting polymer (CP), as a leaky dielectric, is a promising material for high-performance EHDP in order to realize devices with enhanced performance or even wholly new properties and then take a more prominent role in their advanced applications such as organic electronic devices, chemical sensors, field-effect transistors, superhydrophilic/superhydrophobic surfaces, micro-/nanofluidic systems and micro-/nanoelectromechanical systems (MEMS/NEMS).^93–99^ For instance, Rickard *et al.* fabricated well-defined conductive micro-/nano-structures using the thin conducting polymer (*e.g.* polypyrrole, poly(3-hexylthiophene), and so on) films *via* EHDP, as shown in Fig. 4a–f.^100–102^ Moreover, they showed the feasibility of the polypyrrole-based structures with a gate length of 700 nm and a pitch of 500 nm for the electrolyte-gated vertical field-effect transistor (FET) devices, as shown in Fig. 4g–h.

## 5 Fabrication of hierarchical structures

**Fig. 5:**
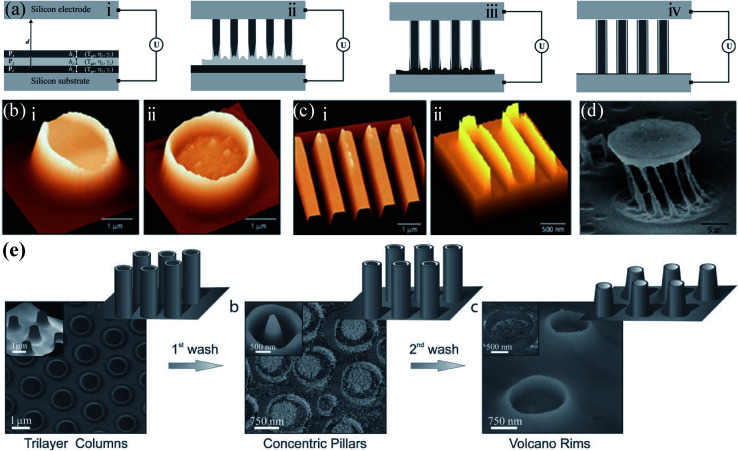
(a) Mechanism of the EHDP structure formation process for multilayer system. Reproduced with permission.^106^ Copyright 2012, WILEY-VCH. (b and c) EHDP with (b) a featureless template and (c) a patterned template patterns of a PMMA-PS-air bilayer: AFM image of a single column before (i) or after (ii) removing the PS core by washing in cyclohexane. Reproduced with permission.^103^ Copyright 2002, Nature Publishing Group. (d) EHDP patterns of a PS-PMMA-air bilayer: SEM image of a single “cage” after removing the PS core by washing in cyclohexane. Reproduced with permission.^105^ Copyright 2006, American Chemical Society. (e) EHDP patterns of an EC-PVA-PVAc-air trilayer: AFM and SEM images of structures derived from (i) the trilayer coaxial pillars, (ii) central PVAc pillars surrounded by thin EC rims, and (iii) EC nanovolcanos. Reproduced with permission.^106^ Copyright 2012, WILEY-VCH.

To date, several techniques structure a single layer of the polymer. For many applications, however, it is desirable to control the spatial arrangement of more than one component. Morariu *et al.* describes a replication process where multiple materials with an air gap between the film and the contactor are processed simultaneously, as shown in Fig. 5a.^103–112^ Using a bilayer or trilayer formed by two or three different polymers, EHDP at both polymer surfaces produce a hierarchic lateral structure that exhibits two or three independent characteristic dimensions, as shown in Fig. 5b–e.^103–112^ This approach might provide a simple strategy for large-area, sub-100 nanometre lithography.

**Fig. 6:**
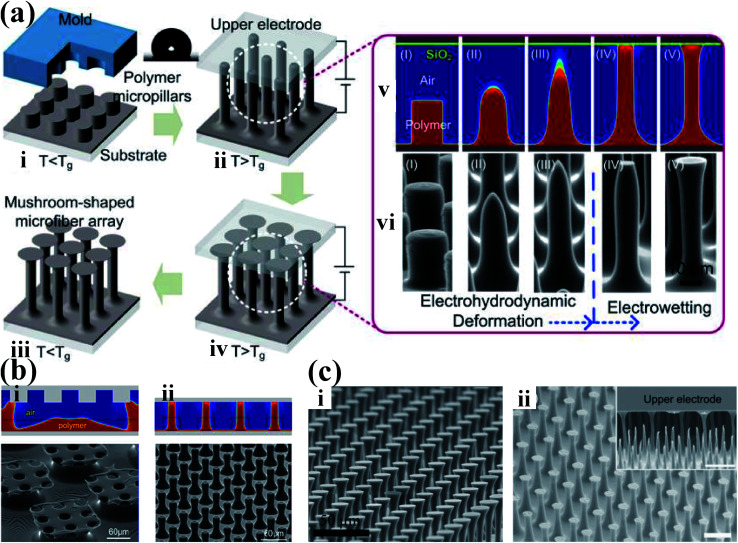
(a) Illustration of a three-step process for fabricating a bioinspired mushroom-shaped microfiber array with a high aspect ratio. Reproduced with permission.^115^ Copyright 2014, American Chemical Society. (b) Simulated and experimental structures fabricated by (i) EHDP-PPT and by (ii) EHDP-PPP. Reproduced with permission.^114^ Copyright 2014, American Chemical Society. (c) The mushroom-shaped micropillars with a well-controlled aspect ratio and tip diameter using various polymers: (i) polymethyl methacrylate (PMMA) and (ii) polyvinylidene fluoride (PVDF). Reproduced with permission.^115^ Copyright 2014, American Chemical Society. Reproduced with permission.^116^ Copyright 2015, Royal Society of Chemistry.

**Fig. 7:**
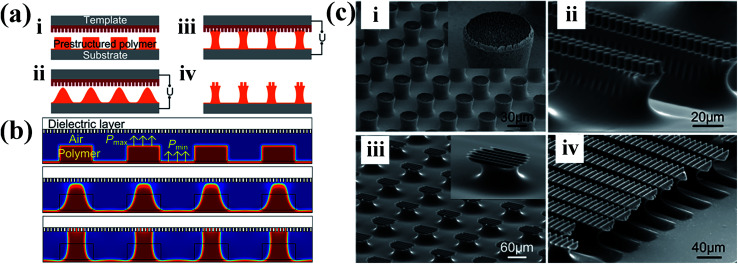
(a) Scheme and (b) numerical simulations of the process for generating hierarchical structures based on electrohydrodynamic (EHD) structure formation with a prestructured polymer. (c) Hierarchically structured polymer fabricated with different patterned template and prestructured polymer: (i) micropillar/nanopillar structure, (ii) micrograting/micropillar structure, (iii) micropillar/micrograting structure, and (iv) micrograting/micrograting structure. Reproduced with permission.^124^ Copyright 2016, American Chemical Society.

EHDP with a featureless template shows the capability to fast and economically create large-scale three-dimensional micro-scale structures on various kinds of materials. However, conventional EHDP with a featureless template can only fabricate a low-aspect-ratio micro-/nanostructures. Therefore, much research have been devoted to increase the aspect ratio of the micro-/nanostructures.^113,114^ For instance, Tian *et al.* developed a novel EHDP technique, EHDP-prepatterned polymer (PPP), to fabricate a hierarchical micro-/nanostructure with a high aspect ratio, as shown in Fig. 6a.^114^ The simulation and experiment showed that EHDP-PPP approach can provide a stronger electric modulation at the same experimental settings, obtaining a hierarchical micro-/nanostructure with a higher aspect ratio compared to EHDP-prepatterned template (PPT), as shown in Fig. 6b. This method can deform various polymers to a mushroom-shaped micropillars with a well-controlled aspect ratio and tip diameter for dry adhesion, nanogenerator, superhydrophilic/superhydrophobic surfaces, microlens arrays, and so on, as shown in Fig. 6c.^115–123^ Furthermore, to fabricate a hierarchically ordered structure, Tian *et al.* improved above EHDP-PPP method by substituting the featureless template with a patterned template which feature size is far less than the characteristic wavelength *λ* of the given system, as shown in Fig. 7a and b. The hierarchically ordered structures with primary and secondary patterns for mass-production, such as, micropillar/nanopillar structure, micrograting/micropillar structure, micropillar/micrograting structure, and micrograting/micrograting structure, were fabricated, as shown in Fig. 7c.^124,125^

**Fig. 8:**
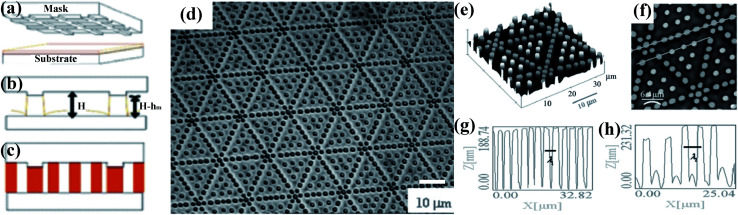
(a–c) The mechanism of the method used to align the pillars. (d) Optical microscopy images of a 45 nm thick PMMA film patterned on a silicon substrate. (e–h) AFM image of a typical pattern formed on the substrate with central pillars taller than those under the ridges. Reproduced with permission.^126^ Copyright 2006, WILEY-VCH.

Russel *et al.* design and utilize the special template patterns to guide pillars into alignment over regions much greater in extent than their natural domain sizes to pattern thin polymer films *via* EHDP, as shown in Fig. 8a–c. Regular rows of pillars form under ridges, and ordered triangular arrays are generated within each individual triangular domain bounded by the ridges, as shown in Fig. 8d. Moreover, the ordered pattern spanned more than 100 periods (400–500 μm), which is the largest array of ordered pillars from EHDP available in the literature. The ordered pattern had two identified characteristic wavelength: one is the spacing between pillars under the ridges, *λ*_1_ (∼2.7 μm), and the other is the spacing between pillars within each small triangular array, *λ*_2_ (∼4.0 μm), as shown in Fig. 8e–h.

**Fig. 9:**
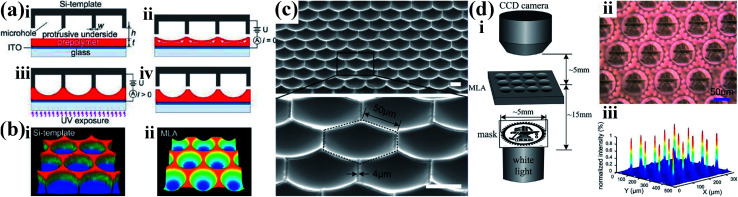
(a) Illustration of EHDP process for generation of concave MLAs. (b) Experimentally measured 3D profiles of (i) the template arrayed with cylindrical microholes and (ii) the corresponding concave MLA, respectively (acquired by LSCM). (c) SEM images of a concave MLA with a hexagonal aperture. Scale bar: 50 μm. (d) Test for the optical properties of concave MLA: (i) diagram of the optical microscopic setup to evaluate imaging and focusing performances of the MLA; (ii) image of the authors' school badge obtained by using a concave MLA with a sag height of ∼12 μm; (iii) measured light intensity profiles of a concave MLA with a sag height of ∼23 μm. Reproduced with permission.^44^ Copyright 2013, American Chemical Society.

Li *et al.* presented an economical method for fabricating a concave microlens arrays (MLAs) with a high quality and high density, as shown in Fig. 9a and b.^44^ The curvature of the MLA can be well-controlled by changing the air gap between the template and polymer film. The MLA has a fill factor calculated as high as up to 93%, as shown in Fig. 9c. Moreover, the MLA has excellent focusing and imaging performances, as shown in Fig. 9d.

**Fig. 10:**
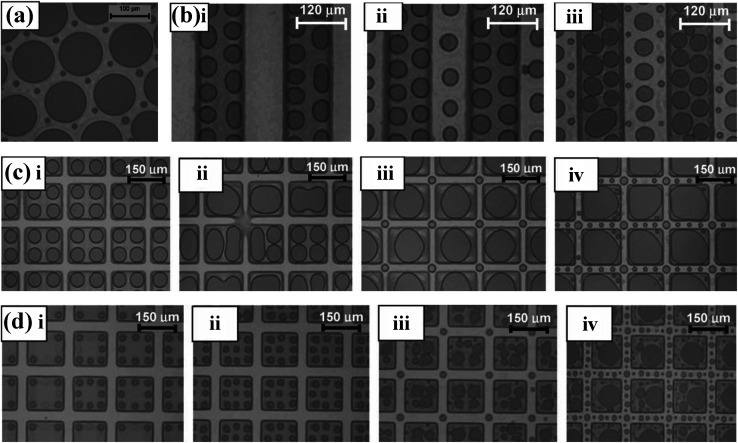
(a) Optical micrographs of EHDP under a top flat electrode using the spatiotemporal modulation of the applied electric field. (b) Optical micrographs of EHDP under a top electrode having parallel ridges and stripes of *w* = 120 μm using the spatiotemporal modulation of the applied electric field. (c) Optical micrographs of EHDP under a box-patterned top electrode using the spatiotemporal modulation of the applied electric field (box dimension ∼2*λ*). (d) Optical micrographs of EHDP under a box-patterned top electrode using the spatiotemporal modulation of the applied electric field (box dimension ∼3*λ*). Reproduced with permission.^26^ Copyright 2011, WILEY-VCH.

Sharma *et al.* showed that the spatiotemporal modulation of the applied electric field influences the pattern morphology in incompletely cross-linked viscoelastic polydimethylsiloxane (PDMS) films, due to the appearance of secondary and tertiary structures, resulting in hierarchical, multiscale patterns, which can be observed in Fig. 10.^26^ Park *et al.* also investigated secondary electrohydrodynamic instability in polymer films by controlling the timescale parameter to produce secondary nanosized patterns between the micrometer-sized grooves.^127^

**Fig. 11:**
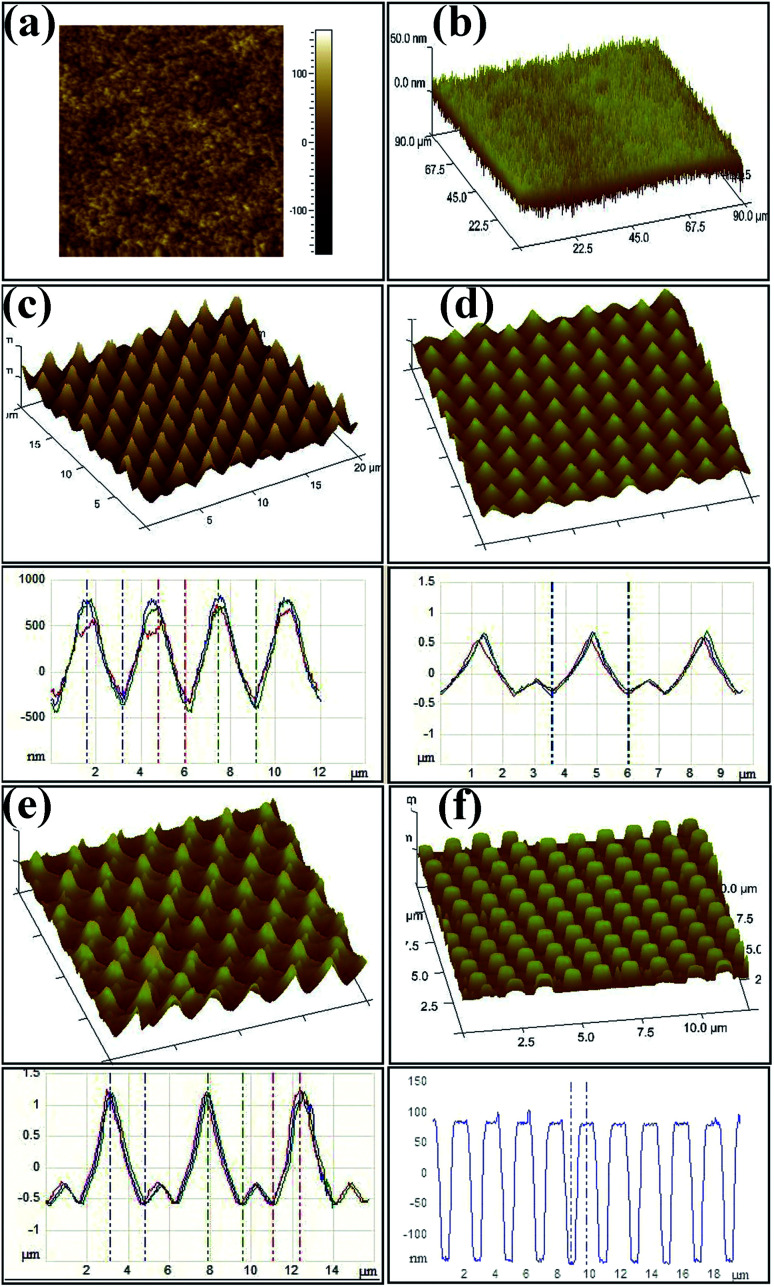
CNT-EHDP replicated patterns. Atomic force microscopy height and three-dimensional images and the corresponding cross-sections of (a) curly nano-hair (CNH) surfaces, (b) straight nano-hairs (SNH), (c) single-level spikes with rounded edges (S1L), (d) two-levelled spiky cones (S2L), (e) two-tiered heretical spiky cones (S2L2) and (f) hexagonal pillars (HP) replicated from the various imposed CNT-based electrodes. Reproduced with permission.^22^ Copyright 2017, Royal Society of Chemistry.

Goldberg-Oppenheimer *et al.* proposed a tunable carbon nanotubes-based electrohydrodynamic patterning (CNT-EHDP) to fabricate unique multiscale structured cones and nanohair-like architectures with various periodicities and dimensions, successfully enabling surface energy minimization, as shown in Fig. 11.^22^ By controlling the hierarchy of micro- to nano cones and spikes, these morphologies provide a range of architectures with sufficient roughness for very low wettability, with the highest contact angle achieved of 173° and their properties can be easily switched between lotus-leaf to rose-petal behavior.

## 6 Aligning and ordering of the anisotropic polymers

**Fig. 12:**
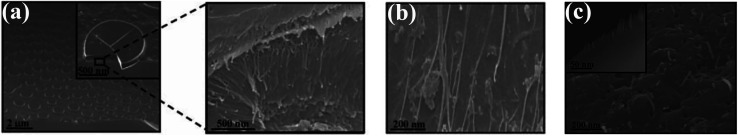
(a) Low- and high-magnification SEM images of the EHDP micro-pillars that contain vertically aligned MWCNTs. (b) SEM images of the EHDP micro-pillars with partial removal of the PS matrix. (c) SEM top-view of the EHDP micro-pillars with partially exposed vertical MWNTs. Inset: SEM images of the EHDP micro-pillars using CNTs which are slightly longer than the pillar height. Reproduced with permission.^128^ Copyright 2011, WILEY-VCH.

In order to take full advantage of the synergistic functions in carbon nanocomposites and hybrids, control of the dispersion, orientation, and interfacial chemistry of carbon materials in the organic or inorganic matrix is required. Moreover, anisotropic composite structures with vertically aligned carbon materials are essential to realize the full potential of carbon materials-based composites both for optimization of their mechanical properties and integration into devices. Goldberg-Oppenheimer *et al.* showed that the EHDP micro-patterns along with the alignment of carbon nanotubes (CNTs) within these patterns can be fabricated for carbon nanotube-polymer nanocomposite films by EHDP with a featureless template, as shown in Fig. 12a and b.^128^ The degree of the carbon nanotube alignment within these patterns can be tuned by adjusting the EHDP parameters. Furthermore, patterned surfaces decorated by CNT brushes can be obtained using either etching techniques or by embedding relatively long nanotubes, as shown in Fig. 12c.

**Fig. 13:**
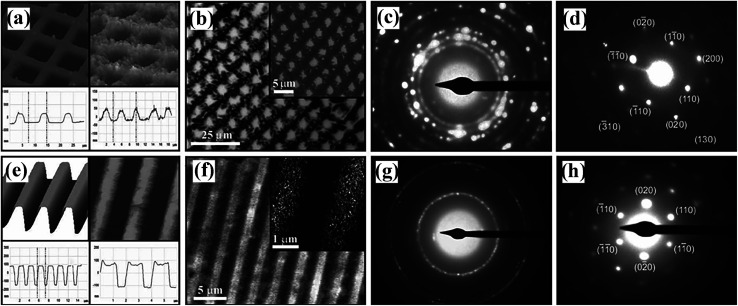
AFM images of a (left) template electrode and (right) the patterns of (a) PCL and (e) PDHA with corresponding cross-sections. Optical micrographs of well-defined crystalline EHDP patterns of (b) PCL and of (f) PDHA. Diffraction patterns for a large area of (c) the PCL and (g) PDHA patterns. Selected area diffraction pattern obtained with an aperture of 3.0 μm of a single PCL crystal in (d) and with a 1.0 μm aperture diameter of a single PDHA crystal in (h). Reproduced with permission.^129^ Copyright 2012, WILEY-VCH.

EHDP enables the structure formation of organic crystalline materials on the micrometer length scale while at the same time exerting control over crystal orientation, as shown in Fig. 13.^129^ Well-ordered structures with nearly vertical walls, comprising stacks of crystals were shown for both PCL (Fig. 13a and b) and PDHA (Fig. 13e and f). A set of nested rings have formed in the diffraction plane for both replicated structures of PCL (Fig. 13c and d) and PDHA (Fig. 13g and h), indicating polycrystalline samples.

**Fig. 14:**
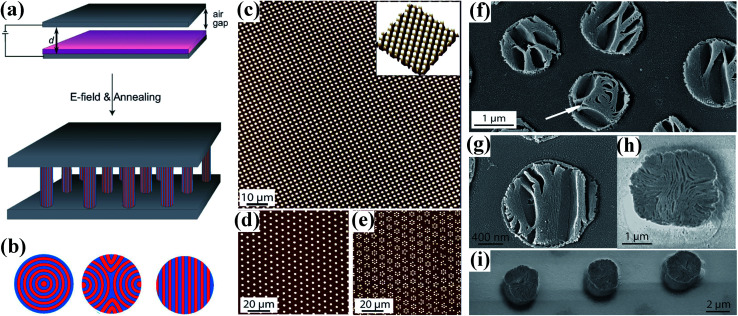
(a) Schematic drawing of the experimental procedure. (b) Three possible in-plane configurations of the lamellar microdomains. (c–e) AFM images of micrometer-sized patterns of PS-*b*-PMMA films made by EHD lithography. (f–i) SEM images of the microphase morphology of PS-*b*-PMMA columns. Reproduced with permission.^131^ Copyright 2008, WILEY-VCH.

EHDP of a block-copolymer (BCP) film also gives rise to hierarchical pattern formation with a micrometer-sized polymer pillars and a 10 nm-scaled microphase morphology in one single step, as shown in Fig. 14.^130–132^ Schematic drawing of the experimental procedure was shown in Fig. 14a. The pattern formation on the micrometer scale of films with two different molecular weights was shown in Fig. 14c–e. The three different in-plane assemblies schematically was shown in Fig. 14b: onion-type concentric alignment of lamellae (Fig. 14f, arrow), parallel sheets (book sheets) (Fig. 14g) and bent lamellae pointing towards the column mantle (Fig. 14h and i). Furthermore, Goldberg-Oppenheimer *et al.* showed a functional block copolymer that contains perylene bismide (PBI) side chains which can crystallize *via* π–π stacking to form an electron conducting microphase is patterned *via* EHDP. The patterned film shows a hierarchical structure with three distinct length scales: micrometer-sized polymer pillars, a 10 nm BCP microphase morphology that is aligned perpendicular to the substrate surface, and a molecular length scale (0.35–3 nm) PBI π–π-stacks traverse the EHDP-generated plugs in a continuous fashion.^133^

## 7 Conclusions and perspective

The review provides an illustrative commentary about the progress and recent developments on EHDP. The key emphasis of the review has been to theoretically and experimentally discuss how the characteristic wavelength and the characteristic time depend on both the external conditions (such as the applied voltage, film thickness, template-substrate spacing) and the initial polymer properties (such as rheological property, electrical property and surface tension). We have theoretically and experimentally discussed various possible strategies for fabricating hierarchical patterns by combining the essential concepts of bottom-up and top-down approaches. Furthermore, we have emphasized aligning and ordering of the anisotropic polymers by EHDP.

Table 1 shows some conventional patterning methods and their characteristics of the resulting patterned surfaces including the cause, the pattern, the size range, the novelty and the limitation. Among the conventional patterning methods, photolithography is a pattern-fabricating technique with high resolution and high throughput, but the photolithography tools are rather expensive and the feature size is limited by optical diffraction limit; nanoimprint is a pattern-transferring technique with low cost, high throughput and high resolution, however, nanoimprint typically require a comparatively large external force to press a patterned template mechanically against the substrate, possibly leading to poor geometrical integrity in the duplicated structure or even to irreversible damage of the template and substrate, making multilayer overlay alignment difficult. EHDP also is a pattern-transferring technique with low cost, high throughput and high resolution. Moreover, the most major advantage of EHDP is the capability to fast and economically create large-scale three-dimensional micro-scale structures on various kinds of materials with featureless templates, even though no long range order and no nano-scale features due to the dielectric breakdown.^71^ In addition, EHDP just need a minimized external force to maintain a proper air gap between the liquid polymer and the template, avoiding the shortcoming of nanoimprint.

**Table 1:** Type of some patterning methods and characteristics of the resulting patterned surfaces

Method	Cause	Pattern	Size range	Novelty	Limitation
Photolithography	Optical diffraction	Pattern of optical diffraction	Nanometer to micrometer	High resolution	High cost
				High throughput	Optical diffraction limit
Nanoimprint	External force	Typically negative replica of template	Nanometer to micrometer	Low cost	Large external force
				High throughput	
				High resolution	
EHDP	External electric field	Typically ordered arrays of pillars, positive replica of template or some hierarchical structures	Submicrometer to micrometer	Low cost	No long range order
				High throughput	No nano-scale features
				High resolution	
				Small external force	

A promising direction of EHDP is the conjugation with other patterning techniques (such as dewetting, nanoimprint, and hot embossing) to fabricate extremely high-aspect-ratio or hierarchical patterns such as, cages, microlens arrays, high-aspect-ratio micropillars, mushroom-shaped micropillars with a well-controlled aspect ratio and tip diameter.^134–137^ In addition, an attractive issue in EHDP is to extend its applicability to a larger class of materials. The benefit of exploiting new kinds of EHD materials lies in that it provides the possibility to not only improve the performance of EHDP, but also integrate functionality into final microstructures because of the interesting physical properties these materials can offer. So a promising direction in EHDP will include the structure formation in functional polymers and their various potential applications.

## Conflicts of interest

The authors declare no competing financial interest.

## Supplementary Material

## References

[cit1] Lai Y.-K., Chen Z., Lin C.-J. (2011). Recent progress on the superhydrophobic surfaces with special adhesion: From natural to biomimetic to functional. J. Nanoeng. Nanomanuf..

[cit2] Chen D., Zhao W., Russell T. P. (2012). P3ht nanopillars for organic photovoltaic devices nanoimprinted by aao templates. ACS Nano.

[cit3] Wang L.-P., Yin K.-Y., Li G., Liu Q., Deng A.-X., Ma H.-Y. (2016). Rhodamine b-loaded star polystyrenes and their luminescent honeycomb-patterned porous films. React. Funct. Polym..

[cit4] Li L., Chen K., Sun L., Xie S., Lin S. (2013). Fabrication of patterned carbon nanotubes with adjustable arrays through controlled mesoscopic dewetting. React. Funct. Polym..

[cit5] Karim M. R., Yeum J. H., Lee M. S., Lim K. T. (2008). Preparation of conducting polyaniline/tio2 composite submicron-rods by the γ-radiolysis oxidative polymerization method. React. Funct. Polym..

[cit6] Chou S. Y., Zhuang L. (1999). Lithographically induced self-assembly of periodic polymer micropillar arrays. J. Vac. Sci. Technol., B.

[cit7] Deshpande P., Chou S. Y. (2001). Lithographically induced self-assembly of microstructures with a liquid-filled gap between the mask and polymer surface. J. Vac. Sci. Technol., B.

[cit8] Deshpande P., Sun X., Chou S. Y. (2001). Observation of dynamic behavior of lithographically induced self-assembly of supramolecular periodic pillar arrays in a homopolymer film. Appl. Phys. Lett..

[cit9] Harkema S., Schäffer E., Morariu M. D., Steiner U. (2003). Pattern replication by confined dewetting. Langmuir.

[cit10] Peng J., Han Y., Yang Y., Li B. (2003). Pattern formation in polymer films under the mask. Polymer.

[cit11] Wu L., Chou S. Y. (2003). Dynamic modeling and scaling of nanostructure formation in the lithographically induced self-assembly and self-construction. Appl. Phys. Lett..

[cit12] Chen L., Zhuang L., Deshpande P., Chou S. (2005). Novel polymer patterns formed by lithographically induced self-assembly (lisa). Langmuir.

[cit13] Wu L., Chou S. Y. (2005). Electrohydrodynamic instability of a thin film of viscoelastic polymer underneath a lithographically manufactured mask. J. Non-Newtonian Fluid Mech..

[cit14] Lin T.-C., Huang L.-C., Huang C.-C., Chao C.-Y. (2011). Formation of self-assembled periodic groovesviathermal drawing lithography foe alignment layers in liquid crystal devices. Soft Matter.

[cit15] Schaffer E., Thurn-Albrecht T., Russell T. P., Steiner U. (2000). Electrically induced structure formation and pattern transfer. Nature.

[cit16] Schaffer E., Thurn-Albrecht T., Russell T. P., Steiner U. (2001). Electrohydrodynamic instabilities in polymer films. Europhys. Lett..

[cit17] Turner L.-A., Downes S., Hill E., Kinloch I. (2014). Investigating the suitability of electrohydrodynamic lithography for the fabrication of cell substrates. J. Mater. Sci..

[cit18] Kathalingam A., Kim H.-S. (2018). Electric field induced pattern formation on pmma and ito layers. Phys. Status Solidi A.

[cit19] Goldberg-Oppenheimer P., Steiner U. (2010). Rapid electrohydrodynamic lithography using low-viscosity polymers. Small.

[cit20] Cheng P. T., Zhou W., Yang F., Lee S. (2019). Growth of polystyrene pillars in electric field. Langmuir.

[cit21] Koupaei A. M., Nazaripoor H., Sadrzadeh M. (2019). Electrohydrodynamic patterning of polyethersulfone membranes. Langmuir.

[cit22] Busa C., Rickard J. J. S., Chun E., Chong Y., Navaratnam V., Goldberg Oppenheimer P. (2017). Tunable superapolar lotus-to-rose hierarchical nanosurfaces *via* vertical carbon nanotubes driven electrohydrodynamic lithography. Nanoscale.

[cit23] Arun N., Sharma A., Shenoy V. B., Narayan K. S. (2006). Electric-field-controlled surface instabilities in soft elastic films. Adv. Mater..

[cit24] Arun N., Sharma A., Pattader P. S., Banerjee I., Dixit H. M., Narayan K. S. (2009). Electric-field-induced patterns in soft viscoelastic films: From long waves of viscous liquids to short waves of elastic solids. Phys. Rev. Lett..

[cit25] Das A. J., Narayan K. S. (2009). Observation of bessel beams from electric-field-induced paterns on polymer surfaces. Opt. Lett..

[cit26] Pattader P. S. G., Banerjee I., Sharma A., Bandyopadhyay D. (2011). Multiscale pattern generation in viscoelastic polymer films by spatiotemporal modulation of electric field and control of rheology. Adv. Funct. Mater..

[cit27] Trease C. H., Foot P. J. S., Augousti A. T. (2017). Electrohydrodynamic patterning in a curable resin over a wide range of fabrication parameters. Eur. Polym. J..

[cit28] Itoh K., Ishida M., Kakinuma Y., Anzai H., Sakirai K. (2019). Development of an electro-adhesive micro pillar array *via* ehd patterning. Smart Mater. Struct..

[cit29] Leach K. A., Lin Z., Russell T. P. (2005). Early stages in the growth of electric field-induced surface fluctuations. Macromolecules.

[cit30] Dickey M. D., Collister E., Raines A., Tsiartas P., Holcombe T., Sreenivasan S. V., Bonnecaze R. T., Willson C. G. (2006). Photocurable pillar arrays formed *via* electrohydrodynamic instabilities. Chem. Mater..

[cit31] Tian H., Wang C., Shao J., Ding Y., Li X. (2014). Electrohydrodynamic pressure enhanced by free space charge for electrically induced structure formation with high aspect ratio. Langmuir.

[cit32] Lv G., Zhang S., Shao J., Tian H., Wang G., Yu D. (2016). Preparation, properties, and efficient electrically induced structure formation of a leaky dielectric photoresist. RSC Adv..

[cit33] Wang G., Lv G., Zhang S., Shao J., Li X., Tian H., Yu D., Zhang L. (2016). A photocurable leaky dielectric for highly electrical insulating electrohydrodynamic micro-/nanopatterns. Soft Matter.

[cit34] Lv G., Zhang S., Shao J., Wang G., Tian H., Yu D. (2017). Rapid fabrication of electrohydrodynamic micro-/nanostructures with high aspect ratio using a leaky dielectric photoresist. React. Funct. Polym..

[cit35] Voicu N. E., Saifullah M. S. M., Subramanian K. R. V., Welland M. E., Steiner U. (2007). Tio2 patterning using electro-hydrodynamic lithography. Soft Matter.

[cit36] Cha S., Lee S., Eun Jang J., Jang A., Pyo Hong J., Lee J., Inn Sohn J., Joon Kang D., Min Kim J. (2013). Ultrafast and low temperature laser annealing for crystalline tio2 nanostructures patterned by electro-hydrodynamic lithography. Appl. Phys. Lett..

[cit37] Lee S., Jung S. H., Kang D. J., Lee J. (2016). Fabrication of a nano-scale pattern with various functional materials using electrohydrodynamic lithography and functionalization. RSC Adv..

[cit38] Chen H., Yu W., Cargill S., Patel M. K., Bailey C., Tonry C., Desmulliez M. P. Y. (2012). Self-encapsulated hollow microstructures formed by electric field-assisted capillarity. Microfluid. Nanofluid..

[cit39] Liu G., Yu W., Li H., Gao J., Flynn D., Kay R. W., Cargill S., Tonry C., Patel M. K., Bailey C., Desmulliez M. P. Y. (2013). Microstructure formation in a thick polymer by electrostatic-induced lithography. J. Micromech. Microeng..

[cit40] Tonry C., Patel M. K., Bailey C., Desmuliez M. P. Y., Cargill S., Yu W. (2013). A method for the micro-encapsulation of dielectric fluids in joined polymer shells. Curr. Org. Chem..

[cit41] Mahajan S., Hutter T., Steiner U., Goldberg Oppenheimer P. (2013). Tunable microstructured surface-enhanced raman scattering substrates *via* electrohydrodynamic lithography. J. Phys. Chem. Lett..

[cit42] Lee S., Jung S., Jang A. R., Hwang J., Shin H. S., Lee J., Kang D. J. (2017). An innovative scheme for sub-50 nm patterning *via* electrohydrodynamic lithography. Nanoscale.

[cit43] Lee Y.-J., Kim Y. W., Kim Y.-K., Yu C.-J., Gwag J. S., Kim J.-H. (2011). Microlens array fabricated using electrohydrodynamic instability and surface properties. Opt. Express.

[cit44] Li X., Tian H., Ding Y., Shao J., Wei Y. (2013). Electrically templated dewetting of a uv-curable prepolymer film for the fabrication of a concave microlens array with well-defined curvature. ACS Appl. Mater. Interfaces.

[cit45] Li R., Wang L., Liu H., Jiang W., Shi Y., Yin L., Zhu Y. (2016). Tunable mirolens array with a large fill-factor: Self-assembly fabrication and electrodrodynamic actuation. Sens. Actuators, A.

[cit46] Harkema S., Steiner U. (2005). Hierarchical pattern formation in thin polymer films using an electric field and vapor sorption. Adv. Funct. Mater..

[cit47] Lv P., You Y., Li J., Zhang Y., Broer D. J., Chen J., Zhou G., Zhao W., Liu D. (2021). Translating 2d director profile to 3d topography in a liquid crystal polymer. Adv. Sci..

[cit48] Wu N., Russel W. B. (2009). Micro- and nano-patterns created *via* electrohydrodynamic instabilities. Nano Today.

[cit49] Voicu N. E., Harkema S., Steiner U. (2006). Electric-field-induced pattern morphologies in thin liquid films. Adv. Funct. Mater..

[cit50] Wu N., Kavousanakis M. E., Russel W. B. (2010). Coarsening in the electrohydrodynamic patterning of thin polymer films. Phys. Rev. E: Stat. Phys., Plasmas, Fluids, Relat. Interdiscip. Top..

[cit51] Amarandei G., O'Dwyer C., Arshak A., Corcoran D. (2013). Fractal patterning of nanoparticles on polymer films and their sers capabilities. ACS Appl. Mater. Interfaces.

[cit52] Tian H., Shao J., Ding Y., Li X., Liu H. (2013). Numerical characterization of electrohydrodynamic micro- or nanopatterning processes based on a phase-field formulation of liquid dielectrophoresis. Langmuir.

[cit53] Tian H., Shao J., Ding Y., Li X., Liu H. (2014). Simulation of polymer rheology in an electrically induced micro- or nano-structuring process based on electrohydrodynamics and conservative level set method. RSC Adv..

[cit54] Lei X., Wu L., Deshpande P., Yu Z., Wu W., Ge H., Chou S. Y. (2003). 100 nm period gratings produced by lithographically induced self-construction. Nanotechnology.

[cit55] Heier J., Groenewold J., Steiner U. (2009). Pattern formation in thin polymer films by spatially modulated electric fields. Soft Matter.

[cit56] Hin T. Y., Liu C., Conway P. P., Yu W., Cargill S., Desmulliez M. P. Y. (2010). Fabrication of a polymeric optical waveguide-on-flex using electrostatic-induced lithography. IEEE Photonics Technol. Lett..

[cit57] Li X., Shao J. Y., Ding Y. C., Tian H. M., Liu H. Z. (2011). Improving the height of replication in ehd patterning by optimizing the electrical properties of the template. J. Micromech. Microeng..

[cit58] Tian H. M., Ding Y. C., Shao J. Y., Li X. M., Liu H. Z. (2013). Formation of irregular micro- or nano-structure with features of varying size by spatial fine-modulation of electric field. Soft Matter.

[cit59] Tian H., Shao J., Chen X., Jiang W., Wang L., Ding Y. (2017). Investigation of the role of template features on the electrically induced structure formation (eisf) for a faithful duplication. Electrophoresis.

[cit60] Tian H., Shao J., Ding Y., Li X., Li X., Liu H. (2011). Influence of distorted electric field distribution on microstructure formation in the electrohydrodynamic patterning process. J. Vac. Sci. Technol., B: Nanotechnol. Microelectron.: Mater., Process., Meas., Phenom..

[cit61] Yang Q., Li B. Q., Ding Y. (2013). Dynamic modelling of micro/nano-patterning transfer by an electric field. RSC Adv..

[cit62] Yang Q., Li B. Q., Ding Y. (2013). A numerical study of nanoscale electrohydrodynamic patterning in a liquid film. Soft Matter.

[cit63] Yang Q., Li B. Q., Ding Y., Shao J. (2014). Steady state of electrohydrodynamic patterning of micro/nanostructures on thin polymer films. Ind. Eng. Chem. Res..

[cit64] Yang Q., Li B. Q., Tian H., Li X., Shao J., Chen X., Xu F. (2016). Deformation hysteresis of electrohydrodynamic patterning on a thin polymer film. ACS Appl. Mater. Interfaces.

[cit65] Boudoire F., Partel S., Toth R., Heier J. (2018). Combining parallel pattern generation of electrohydrodynamic lithography with serial addressing. RSC Adv..

[cit66] Goldberg-Oppenheimer P. , Electrohydrodynamic patterning of functional materials, Doctoral thesis, The University of Cambridge, Springer Theses, 2013

[cit67] Pease III L. F., Russel W. B. (2002). Linear stability analysis of thin leaky dielectric films subjected to electric fields. J. Non-Newtonian Fluid Mech..

[cit68] Pease III L. F., Russel W. B. (2003). Electrostatically induced submicron patterning of thin perfect and leaky dielectric films: A generalized linear stability analysis. J. Chem. Phys..

[cit69] Pease III L. F., Russel W. B. (2004). Limitations on length scales for electrostatically induced submicrometer pillars and holes. Langmuir.

[cit70] Pease III L. F., Russel W. B. (2006). Charge driven, electrohydrodynamic patterning of thin films. J. Chem. Phys..

[cit71] Lau C. Y., Russel W. B. (2011). Fundamental limitations on ordered electrohydrodynamic patterning. Macromolecules.

[cit72] Hwang J., Park H., Lee J., Kang D. J. (2021). Parametric scheme for rapid nanopattern replication *via* electrohydrodynamic instability. RSC Adv..

[cit73] Tomar G., Shankar V., Sharma A., Biswas G. (2007). Electrohydrodynamic instability of a confined viscoelastic liquid film. J. Non-Newtonian Fluid Mech..

[cit74] Sarkar J., Sharma A., Shenoy V. B. (2008). Electric-field induced instabilities and morphological phase transitions in soft elastic films. Phys. Rev. E: Stat. Phys., Plasmas, Fluids, Relat. Interdiscip. Top..

[cit75] Roy P., Gooh Pattader P. S. (2020). Electrohydrodynamic instability: Effect of rheological characteristics on the morphological evolution of liquid crystal–polymer interface. Bull. Mater. Sci..

[cit76] Tsiartas P. C., Dickey M. D., Allrich K. E., Willson C. G. (2006). Photocurable Pillar Arrays Formed *via* AC- and Ultrasound-Induced Electrohydrodynamic Instabilities. Proc. of SPIE.

[cit77] Lin Z., Kerle T., Baker S. M., Hoagland D. A., Schäffer E., Steiner U., Russell T. P. (2001). Electric field induced instabilities at liquid/liquid interfaces. J. Chem. Phys..

[cit78] Bae J., Glogowski E., Gupta S., Chen W., Emrick T., Russell T. P. (2008). Effect of nanoparticles on the electrohydrodynamic instabilities of polymer/nanoparticle thin films. Macromolecules.

[cit79] Lin Z., Kerle T., Russell T. P., Schaffer E., Steiner U. (2002). Structure formation at the interface of liquid/liquid bilayer in electric field. Macromolecules.

[cit80] Kho D. H., Chae S. H., Jeong U., Kim H. Y., Kim J. K. (2005). Morphological development at the interface of polymer/polymer bilayer with an *in situ* compatibilizer under electric field. Macromolecules.

[cit81] Thaokar R. M., Kumaran V. (2005). Electrohydrodynamic instability of the interface between two fluids confined in a channel. Phys. Fluids.

[cit82] Wu N., Russel W. B. (2006). Electrohydrodynamic instability of dielectric bilayers: Kinetics and thermodynamics. Ind. Eng. Chem. Res..

[cit83] John K., Hänggi P., Thiele U. (2008). Ratchet-driven fluid transport in bounded two-layer films of immiscible liquids. Soft Matter.

[cit84] Uguz A. K., Ozen O., Aubry N. (2008). Electric field effect on a two-fluid interface instability in channel flow for fast electric times. Phys. Fluids.

[cit85] Gambhire P., Thaokar R. M. (2011). Linear stability analysis of electrohydrodynamic instabilities at fluid interfaces in the “small feature” limit. Eur. Phys. J. E.

[cit86] Bae J. (2012). Electrohydrodynamic instabilities of polymer thin films: Filler effect. J. Ind. Eng. Chem..

[cit87] Gambhire P., Thaokar R. M. (2012). Role of conductivity in the electrohydrodynamic patterning of air-liquid interfaces. Phys. Rev. E: Stat., Nonlinear, Soft Matter Phys..

[cit88] Nazaripoor H., Koch C. R., Sadrzadeh M., Bhattacharjee S. (2015). Electrohydrodynamic patterning of ultra-thin ionic liquid films. Soft Matter.

[cit89] Nazaripoor H., Koch C. R., Sadrzadeh M., Bhattacharjee S. (2016). Compact micro/nano electrohydrodynamic patterning: Using a thin conductive film and a patterned template. Soft Matter.

[cit90] Mondal K., Bandyopadhyay D. (2014). Electro-capillary instabilities of thin leaky elastic-viscous bilayers. Phys. Fluids.

[cit91] Nazaripoor H., Koch C. R., Bhattacharjee S. (2014). Electrical perturbations of ultrathin bilayers: Role of ionic conductive layer. Langmuir.

[cit92] Shankar V., Sharma A. (2004). Instability of the interface between thin fluid films subjected to electric fields. J. Colloid Interface Sci..

[cit93] Jiang L., Wang X., Chi L. (2011). Nanoscaled surface patterning of conducting polymers. Small.

[cit94] Wang J. Z., Zheng Z. H., Li H. W., Huck W. T., Sirringhaus H. (2004). Dewetting of conducting polymer inkjet droplets on patterned surfaces. Nat. Mater..

[cit95] Li L., Hirtz M., Wang W., Du C., Fuchs H., Chi L. (2010). Patterning of polymer electrodes by nanoscratching. Adv. Mater..

[cit96] Kim B. H., Onses M. S., Lim J. B., Nam S., Oh N., Kim H., Yu K. J., Lee J. W., Kim J. H., Kang S. K., Lee C. H., Lee J., Shin J. H., Kim N. H., Leal C., Shim M., Rogers J. A. (2015). High-resolution patterns of quantum dots formed by electrohydrodynamic jet printing for light-emitting diodes. Nano Lett..

[cit97] Huang J., Virji S., Weiller B. H., Kaner R. B. (2003). Polyaniline nanofibers: Facile synthesis and chemical sensors. J. Am. Chem. Soc..

[cit98] Liu H., Kameoka J., Czaplewski D. A., Craighead H. G. (2004). Polymeric nanowire chemical sensor. Nano Lett..

[cit99] Dong B., Zhong D. Y., Chi L. F., Fuchs H. (2005). Patterning of conducting polymers based on a random copolymer strategy: Toward the facile fabrication of nanosensors exclusively based on polymers. Adv. Mater..

[cit100] Rickard J. J., Farrer I., Goldberg Oppenheimer P. (2016). Tunable nanopatterning of conductive polymers *via* electrohydrodynamic lithography. ACS Nano.

[cit101] Manigandan S., Majumder S., Suresh A., Ganguly S., Kargupta K., Banerjee D. (2010). Electric field induced dewetting and pattern formation in thin conducting polymer film. Sens. Actuators, B.

[cit102] Ye Z., Cui H., Yang X., Qiu F. (2015). Charge injection promoted electrohydrodynamic instabilities in poly(3-hexylthiophene) thin films. J. Mater. Chem. C.

[cit103] Morariu M. D., Voicu N. E., Schaffer E., Lin Z., Russell T. P., Steiner U. (2003). Hierarchical structure formation and pattern replication induced by an electric field. Nat. Mater..

[cit104] Leach K. A., Gupta S., Dickey M. D., Willson C. G., Russell T. P. (2005). Electric field and dewetting induced hierarchical structure formation in polymer/polymer/air trilayers. Chaos.

[cit105] Dickey M. D., Gupta S., Leach K. A., Collister E., Willson C. G., Russell T. P. (2006). Novel 3-d structures in polymer films by coupling external and internal fields. Langmuir.

[cit106] Goldberg-Oppenheimer P., Mahajan S., Steiner U. (2012). Hierarchical electrohydrodynamic structures for surface-enhanced raman scattering. Adv. Mater..

[cit107] Reddy P. D. S., Bandyopadhyay D., Sharma A. (2010). Self-organized ordered arrays of core-shell columns in viscous bilayers formed by spatially varying electric fields. J. Phys. Chem. C.

[cit108] Reddy P. D. S., Bandyopadhyay D., Sharma A. (2012). Electric-field-induced instabilities in thin liquid trilayers confined between patterned electrodes. J. Phys. Chem. C.

[cit109] Bandyopadhyay D., Sharma A., Thiele U., Reddy P. D. (2009). Electric-field-induced interfacial instabilities and morphologies of thin viscous and elastic bilayers. Langmuir.

[cit110] Roberts S. A., Kumar S. (2010). Electrohydrodynamic instabilities in thin liquid trilayer films. Phys. Fluids.

[cit111] Bandyopadhyay D., Dinesh Sankar Reddy P., Sharma A. (2012). Electric field and van der waals force induced instabilities in thin viscoelastic bilayers. Phys. Fluids.

[cit112] Mondal K., Kumar P., Bandyopadhyay D. (2013). Electric field induced instabilities of thin leaky bilayers pathways to uniyue morphologies and miniaturization. J. Chem. Phys..

[cit113] Dickey M. D., Raines A., Collister E., Bonnecaze R. T., Sreenivasan S. V., Willson C. G. (2008). High-aspect ratio polymeric pillar arrays formed *via* electrohydrodynamic patterning. J. Mater. Sci..

[cit114] Tian H. M., Shao J. Y., Ding Y. C., Li X. M., Hu H. (2014). Electrohydrodynamic micro-/nanostructuring processes based on prepatterned polymer and prepatterned template. Macromolecules.

[cit115] Hu H., Tian H., Li X., Shao J., Ding Y., Liu H., An N. (2014). Biomimetic mushroom-shaped microfibers for dry adhesives by electrically induced polymer deformation. ACS Appl. Mater. Interfaces.

[cit116] Chen X., Tian H., Li X., Shao J., Ding Y., An N., Zhou Y. (2015). A high performance p(vdf-trfe) nanogenerator with self-connected and vertically integrated fibers by patterned ehd pulling. Nanoscale.

[cit117] Chen X., Shao J., Li X., Tian H. (2016). A flexible piezoelectric-pyroelectric hybrid nanogenerator based on p(vdf-trfe) nanowire array. IEEE Trans. Nanotechnol..

[cit118] Hu H., Shao J., Tian H., Li X., Jiang C. (2016). Mushroom-shaped micropillars for robust nonwetting surface by electrohydrodynamic structuring technique. IEEE Trans. Nanotechnol..

[cit119] Lv G., Liu Y., Shao J., Tian H., Yu D. (2018). Facile fabrication of electrohydrodynamic micro-/nanostructures with high aspect ratio of a conducting polymer for large-scale superhydrophilic/superhydrophobic surfaces. Macromol. Mater. Eng..

[cit120] Tian H., Shao J., Chen X., Wang L., Ding Y. (2017). A versatile approach to fabricate modulated micro-/nanostructures by electrohydrodynamic structuring on prepatterned polymer. J. Micromech. Microeng..

[cit121] Lv G., Tian H., Shao J., Yu D. (2019). Synthesis, characterization, and efficient electrohydrodynamic patterning with a high aspect ratio of a soluble oligomerpyrrole derivative. React. Funct. Polym..

[cit122] Tian H., Shao J., Hu H., Wang L., Ding Y. (2016). Role of space charges inside a dielectric polymer in the electrohydrodynamic structure formation on a prepatterned polymer (esf-pp). RSC Adv..

[cit123] Hu H., Tian H., Shao J., Ding Y., Jiang C., Liu H. (2014). Fabrication of bifocal microlens arrays based on controlled electrohydrodynamic reflowing of pre-patterned polymer. J. Micromech. Microeng..

[cit124] Tian H., Shao J., Hu H., Wang L., Ding Y. (2016). Generation of hierarchically ordered structures on a polymer film by electrohydrodynamic structure formation. ACS Appl. Mater. Interfaces.

[cit125] Lv G., Hu X., Hao L., Tian H., Shao J., Yu D. (2021). Facile fabrication of a flexible patterned film with diverse micro-/nanostructures *via* electrohydrodynamic patterning. Ind. Eng. Chem. Res..

[cit126] Wu N., Pease L. F., Russel W. B. (2006). Toward large-scale alignment of electrohydrodynamic patterning of thin polymer films. Adv. Funct. Mater..

[cit127] Park H., Hwang J., Lee T. H., Lee J., Kang D. J. (2021). Fog collection based on secondary electrohydrodynamic-induced hybrid structures with anisotropic hydrophilicity. ACS Appl. Mater. Interfaces.

[cit128] Goldberg-Oppenheimer P., Eder D., Steiner U. (2011). Carbon nanotube alignment *via* electrohydrodynamic patterning of nanocomposites. Adv. Funct. Mater..

[cit129] Goldberg-Oppenheimer P., Kohn P., Langford R. M., Steiner U. (2012). Patterning of crystalline organic materials by electro-hydrodynamic lithography. Small.

[cit130] Xiang H., Lin Y., Russell T. P. (2004). Electrically induced patterning in block copolymer films. Macromolecules.

[cit131] Voicu N. E., Ludwigs S., Steiner U. (2008). Alignment of lamellar block copolymers *via* electrohydrodynamic-driven micropatterning. Adv. Mater..

[cit132] Zhou Y., Nicolas A., Thomas K. R., Steiner U. (2012). Interplay of electrohydrodynamic structure formation and microphase alignment in lamellar block copolymers. Soft Matter.

[cit133] Goldberg-Oppenheimer P., Kabra D., Vignolini S., Hüttner S., Sommer M., Neumann K., Thelakkat M., Steiner U. (2013). Hierarchical orientation of crystallinity by block-copolymer patterning and alignment in an electric field. Chem. Mater..

[cit134] Lee S. H., Kim P., Jeong H. E., Suh K. Y. (2006). Electrically induced formation of uncapped, hollow polymeric microstructures. J. Micromech. Microeng..

[cit135] Dwivedi S., Vivek, Mukherjee R., Atta A. (2017). Formation and control of secondary nanostructures in electro-hydrodynamic patterning of ultra-thin films. Thin Solid Films.

[cit136] Dwivedi S., Narayanan R., Chaudhary R., Mukherjee R., Atta A. (2018). Controlled nanoscale electrohydrodynamic patterning using mesopatterned template. ACS Omega.

[cit137] Li H., Yu W., Wang Y., Bu H., Liu Z., Abraham E., Desmulliez M. P. Y. (2014). Simulation of the electrohydrodynamic instability process used in the fabrication of hierarchic and hollow micro/nanostructures. RSC Adv..

